# The short wavelength electro-oculogram (SW-EOG) in best disease: preliminary results

**DOI:** 10.1007/s10633-026-10081-2

**Published:** 2026-02-02

**Authors:** Srikanta Kumar Padhy, Paul A. Constable

**Affiliations:** 1 Anant Bajaj Retina Institute, LV Prasad Eye Institute, Mithu Tulsi Chanrai Campus, Bhubaneswar, 751024 India; 2 College of Nursing and Health Sciences, Flinders University, Caring Futures Institute, Adelaide, Australia

**Keywords:** Bestrophin, Light-rise, Blue light, Clinical protocol

## Abstract

**Purpose:**

The clinical electrooculogram (EOG) is used for the diagnosis of bestrophinopathies to evaluate the function of the retinal pigment epithelium. The current ISCEV test protocol uses a broad band white light with luminance of 100 cd/m^2^. An alternative monochromatic 448 nm short wavelength (SW-EOG) has been proposed but to date has not been evaluated in clinical cases where the standard EOG is abnormal.

**Methods:**

To evaluate the clinical potential of the SW-EOG four genetically confirmed cases of Best Vitelliform Macular Dystrophy were tested using the standard white (100 cd/m^2^) and SW-EOG (448 nm) at 30 cd/m^2^ to ascertain if the SW-EOG was also affected. In addition, a qualitative 5-point Likert survey was conducted to gauge overall patient comfort.

**Results:**

In all four cases the SW-EOG was reduced and provided an equivalent clinical measure for RPE dysfunction. All four participants rated the SW-EOG as being more comfortable than the white standard EOG test.

**Conclusions:**

This is the first demonstration of an alternative stimulus for the EOG that provided clinically valid results with greater comfort than the current ISCEV protocol. Further studies are required to validate the SW-EOG as an alternative to the white broad band stimulus.

## Introduction

Best vitelliform macular dystrophy (BVMD, Best’s disease) is an autosomal dominant maculopathy caused by mutations in the *BEST1* gene encoding a Ca^2^⁺-activated chloride channel in the retinal pigment epithelium (RPE) [1]. A hallmark of BVMD is a markedly abnormal clinical electrooculogram (EOG): the light-peak to dark-trough ratio (LP:DT_ratio_) is universally reduced (usually ≤ 1.5) in affected patients and even in phenotypically normal carriers [2, 3]. By contrast, the full-field electroretinogram (ERG) is often normal, since bestrophinopathy primarily disrupts RPE ionic transport rather than photoreceptor function. The ISCEV standard EOG uses a 100 cd/m^2^ white Ganzfeld light stimulus, yielding a normal LP:DT_ratio_ typically between 1.7 and 4.3 with a LP time ranging from 7 to 12 min [4, 5] and a median value of 2.35 based on a meta-anlaysis of studies [6]. In BVMD, impaired RPE function causes a severely attenuated light-rise: adults typically have ratios well below normal, and pediatric Best cases often have virtually absent light-rise (e.g. Arden ≈1.0–1.5) with Padhy et al. [7] reporting a mean LP:DT_ratio_ of 1.40 ± 0.29 in children with Best disease, with ~ 27% showing no detectable light rise. Clinically, a LP:DT_ratio_ of < 1.65 is considered diagnostic for Best’s disease, and the EOG remains the gold-standard functional test for BVMD.

Despite its diagnostic utility, the standard 100 cd/m^2^ white flash can be visually uncomfortable following 15 min of dark adaptation. Recent studies have therefore examined monochromatic stimuli as alternatives. Constable and Kapoor [8] showed that a blue color LED (448 nm, 30 cd/m^2^) produced an equivalent LP:DT_ratio_ and timing in normal subjects as the ISCEV white standard, while greatly reducing photophobia. In that study the median LP:DT_ratio_ was 2.49 (white) vs 2.47 (448 nm) with no significant difference (p = 0.99), and peak times were equivalent (~ 9 vs 8 min). Other monochromatic wavelengths were also examined at 30 cd/m^2^ including (534, 596 and 634 nm) which also elicited a ‘light-rise’ with median LP:DT_ratios_ ranging from 2.03 to 2.36 and so were not the best candidates to use as an alternative to the standard broad wavelength 100 cd/m^2^ white stimulus [8]. Importantly, subjects reported the SW stimulus to be more comfortable than the bright white light. These findings suggested that a blue stimulus or SW-EOG could offer an alternative to the higher luminance white light currently recommended in the ISCEV standard [4]. The findings of Constable and Kapoor (2021) confirmed earlier studies on the wavelength dependence of the light-rise shows greater sensitivity to short and medium length wavelengths [9–11].

The mechanism by which a SW-EOG occurs implies either the 448 nm stimulus initiates the release of a the same ‘light-rise’ substance from the rods that initiates a rise in intracellular Ca^2+^ in the RPE and depolarizes the basolateral membrane as is the standard explanation for the light-rise of the EOG [12–17]. Certainly, the 448 nm SW-EOG may well trigger the same release of a light rise substance from the rods as one possible mechanism. It is unlikely that the response is driven by the less numerous s-cones in the retina although a cone contribution to the EOG has been proposed [10, 11]. Contributions from melanopsin have also been proposed and excluded with knock out mouse exhibiting normal DC-ERG responses [8]. A non-photoreceptor origin of the light-rise has also been suggested with light triggering the metabolism of inositol tri-phosphate in the RPE apical membrane which may occur following the SW stimulus but will require further studies to verify this pathway [18]. The light rise substance may be ATP released by photoreceptors as proposed by Schreiber and Kunzelmann (2016) [19], although an alternative pathway may be that ATP is trafficked from the RPE through vesicular release in response to cellular stress or volume increase which initiates the light rise through activation of purinergic apical receptors [20, 21]. The SW stimulus may evoke release of ATP through this hypothetical pathway.

Irrespective of the mechanism the aim of this preliminary report is to assess the potential clinical utility of the SW-EOG. This case series uses EOGs in four genetically confirmed BVMD patients to compare the ISCEV-standard white light (100 cd/m^2^) to the SW-EOG using 448 nm monochromatic LED with 30 cd/m^2^ luminance. The aim was to compare the LP:DT_ratios_ and timing between stimuli, assess any impact on diagnostic accuracy, and record patient comfort.

## Methods

Subjects: Four patients (aged 12–46, mean 28 years; 3 male, 1 female) with clinical and genetic confirmation of BVMD (mutations in *BEST1*) were recruited. Each had characteristic bilateral vitelliform lesions and normal full-field ERGs. Each had characteristic bilateral vitelliform lesions and normal full-field ERGs. All procedures adhered to the Declaration of Helsinki with ethical approval from the LV Prasad eye Institute’s human research ethics committee (Approval No. 2025–214-BHR-14). Pupils were dilated (tropicamide 1%) prior to testing. Participants were examined at the LV Prasad Eye Institute, Bhubaneswar, India.

EOG Recording: EOGs were performed with a Ganzfeld stimulator (MonColor, Metrovision, Pérenchies, France) and Burian-Allen electrodes following ISCEV guidelines [4]. After dark adaptation (15 min), standing potential was measured via alternate gaze every minute for 20 min under continuous light. Two conditions were tested in each patient on separate days (randomized order): (1) White light: standard ISCEV protocol, white (0.33,0.33) at 100 cd/m^2^. (2) SW light: monochromatic 448 nm LED (0.166, 0.018) at 30 cd/m^2^. The lower luminance of the SW stimulus (30 vs 100) matched Constable and Kapoor [8]. The spectral output was verified with a photometer and ensured equal Ganzfeld uniformity. In both conditions, the DT was defined as the minimum amplitude (µV) in the dark phase and the as the maximum after light onset. The LP:DT_ratio_ was calculated (typically reported as a ratio or percentage). We also recorded the time to LP (minutes from light onset). Subjective Comfort: Immediately after each test, patients rated light discomfort on a 0–5 scale (0 = no discomfort, 5 = severe).

LP:DT_ratios_ and time-to-peak were compared between the white and SW conditions for each patient. Given the small sample, results are described qualitatively. A comparison with normative values (white stimulus LP:DT_ratio_ 2.28–2.42 in healthy adults was made to illustrate the degree of abnormality [5].

## Results

**Fig. 1:**
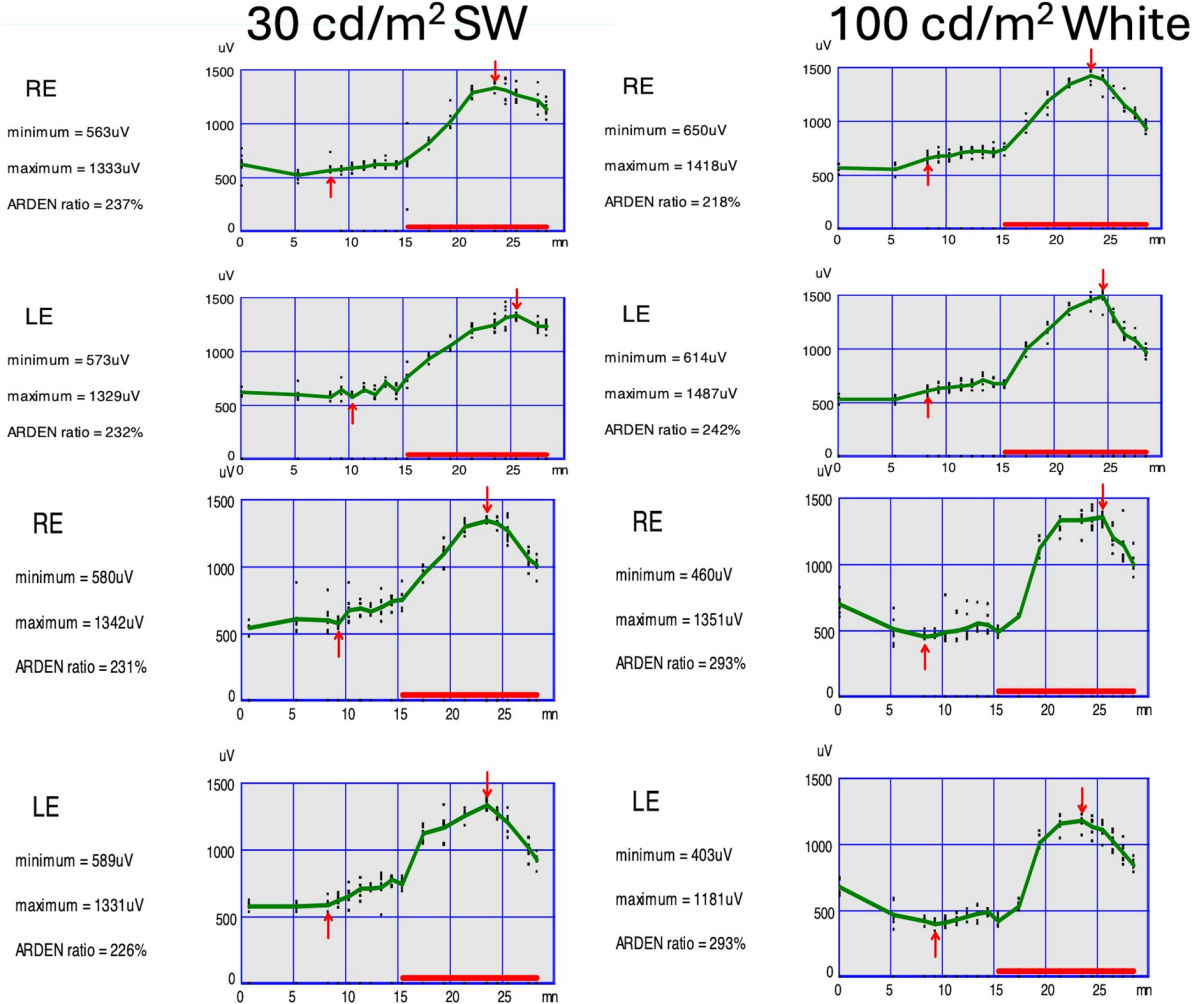
Representee electrooculogram plots for two normal subjects recorded in each eye using a 30 cd/m^2^ short wavelength (SW) 448 nm luminance after 15 min dark adaptation (left panels). The right panels show the same subject’s electrooculogram recorded using a 100 cd/m^2^ broad band white luminance. “Arden” or LP: DT_ratios_ for each condition are shown as a percentage

**Fig. 2:**
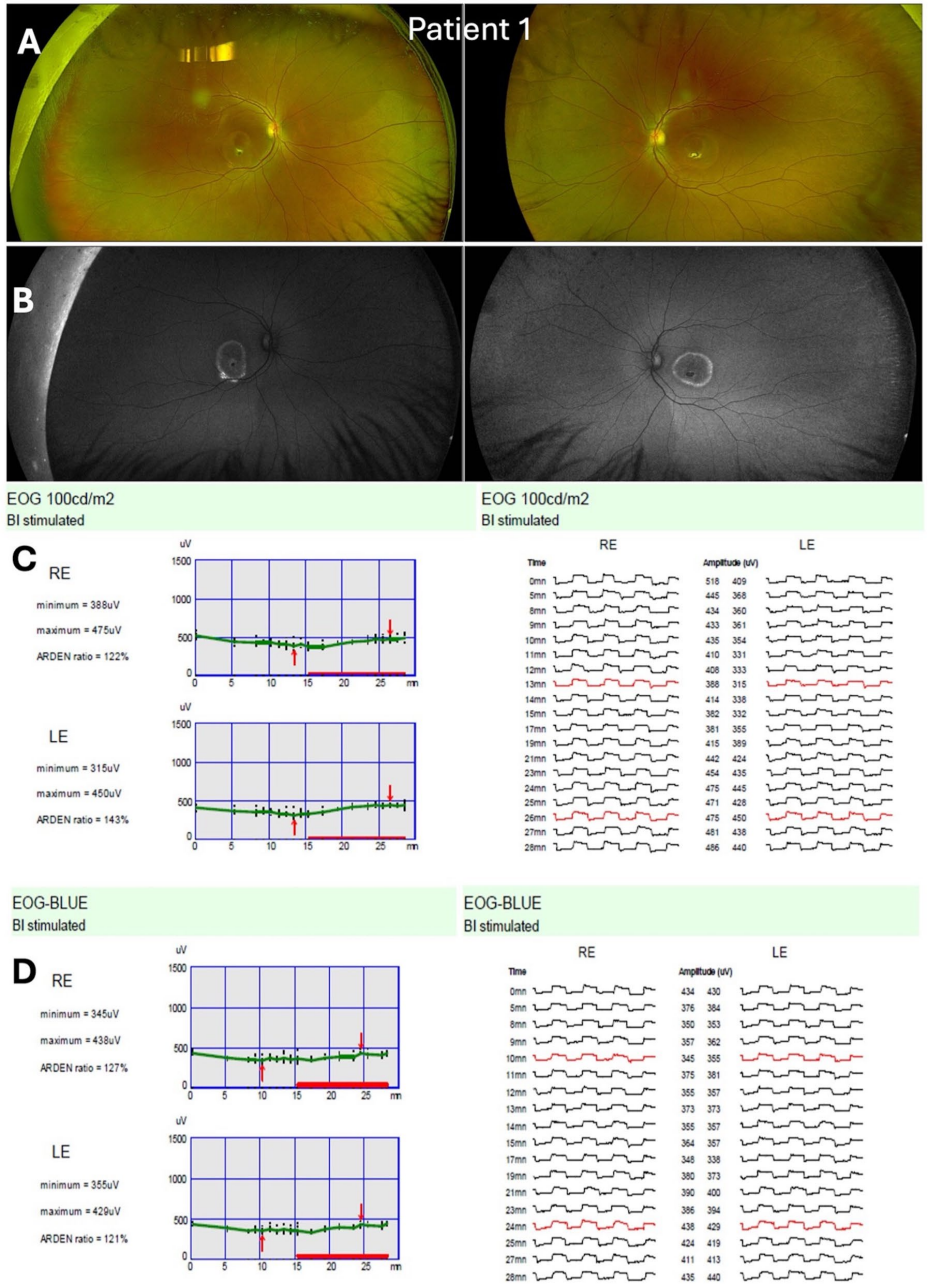
Patient 1 with Best disease (pseudohypopyon stage, both eyes). Panel (**A**) shows wide-field fundus photographs, and Panel (**B**) the corresponding wide-field fundus autofluorescence images. The hallmark finding of Best disease is a bilateral macular lesion with hyperfluorescent lipofuscin. Panel (**C**) depicts the ISCEV standard white light EOG (100 cd/m^2^), and Panel (**D**) the short-wave EOG (SW-EOG) recorded with a 30 cd/m^2^ LED (peak emission at 448 nm). Both recordings demonstrate reduced responses, with ‘Arden’ LP:DT_ratios_ reported as percentages for the right eye (RE) and left eye (LE). Genetic testing revealed a *BEST1* variant, Exon 4 *c.287A* > *T* (*p.Gln96Leu*, heterozygous), classified as likely pathogenic

**Fig. 3:**
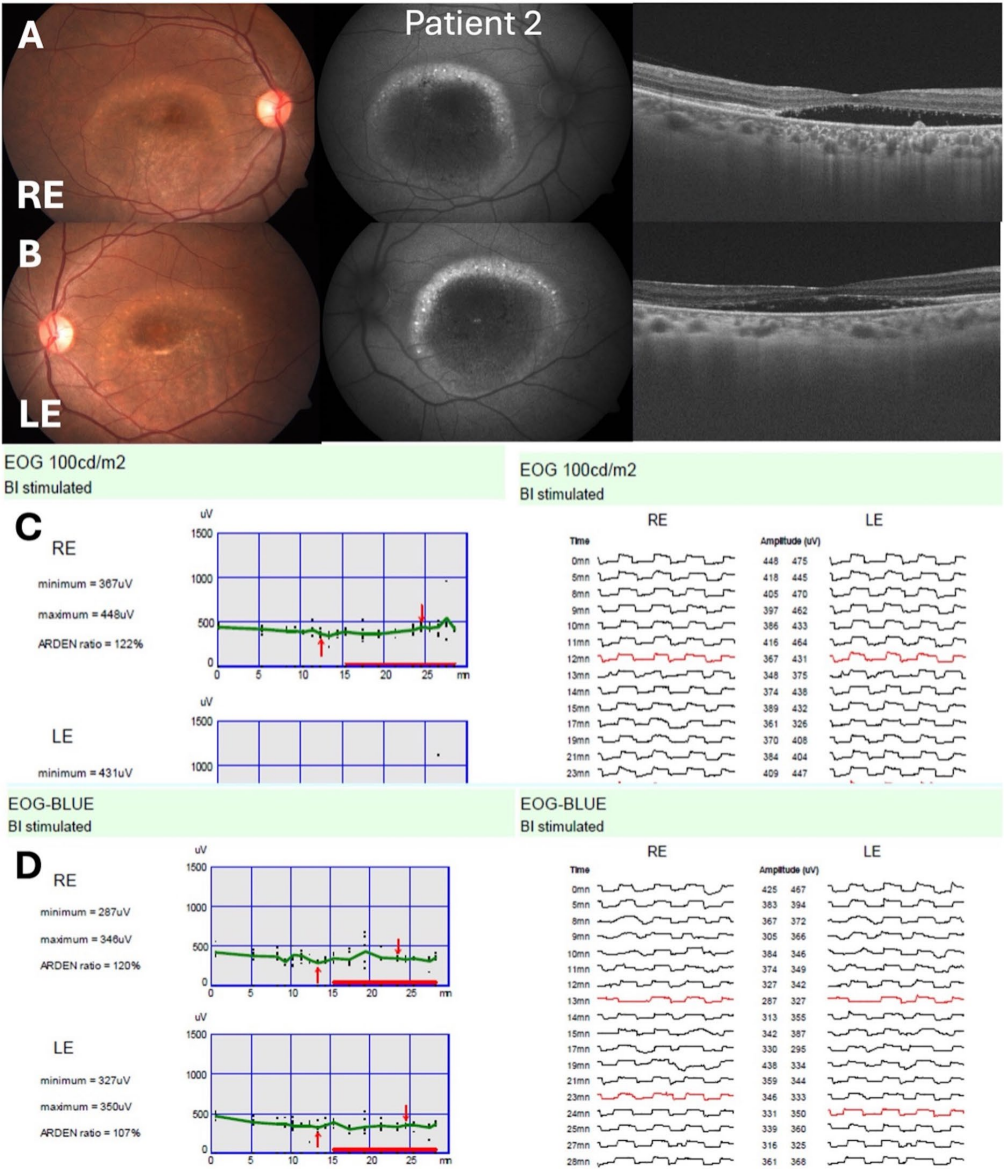
Patient 2 with Best disease (pseudohypopyon stage, both eyes). Panel (**A**) (RE) and Panel (**B**) (LE) display color fundus images, fundus autofluorescence, and optical coherence tomography (OCT). The characteristic bilateral macular lesion is seen, consisting of hyperfluorescent lipofuscin and a vitelliform deposit at the macula. Panel (**C**) illustrates the ISCEV standard white light EOG (100 cd/m^2^), and Panel (**D**) the SW-EOG (30 cd/m^2^ LED, 448 nm peak emission). Both recordings show reduced responses, with ‘Arden’ LP:DT_ratios_ reported for the RE and LE. Genetic analysis identified two *BEST1* variants, Exon 4 *c.280 T* > *C (p.Trp94Arg)* and Exon 7 *c.857G* > *T (p.Gly286Val)*, both heterozygous and of likely pathogenic

**Fig. 4:**
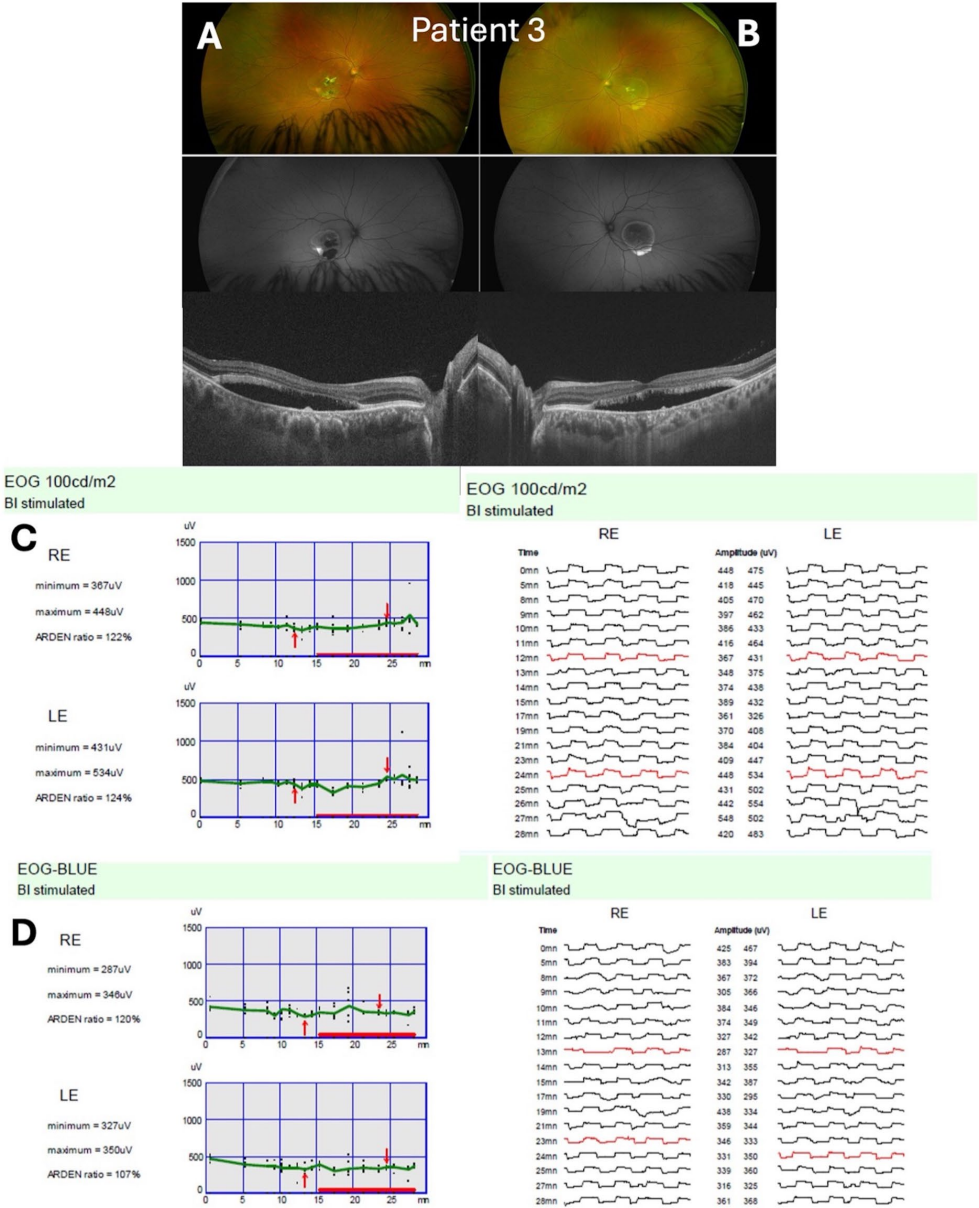
Patient 3 with Best disease (pseudohypopyon stage, both eyes). Panel (**A**) (RE) and Panel (**B**) (LE) show wide-field fundus, fundus autofluorescence, and OCT images, demonstrating bilateral macular lesions with hyperfluorescent lipofuscin and vitelliform changes. Panel (**C**) presents the ISCEV standard white light EOG (100 cd/m^2^), and Panel (**D**) the SW-EOG (30 cd/m^2^ LED, 448 nm peak emission). Both eyes show reduced responses, with ‘Arden’ LP: DT_ratios_ reported for the RE and LE. Genetic testing revealed a *BEST1* variant, Exon 3 c*.217A* > *T (p.Ile73Phe*, heterozygous), of likely pathogenic

**Fig. 5:**
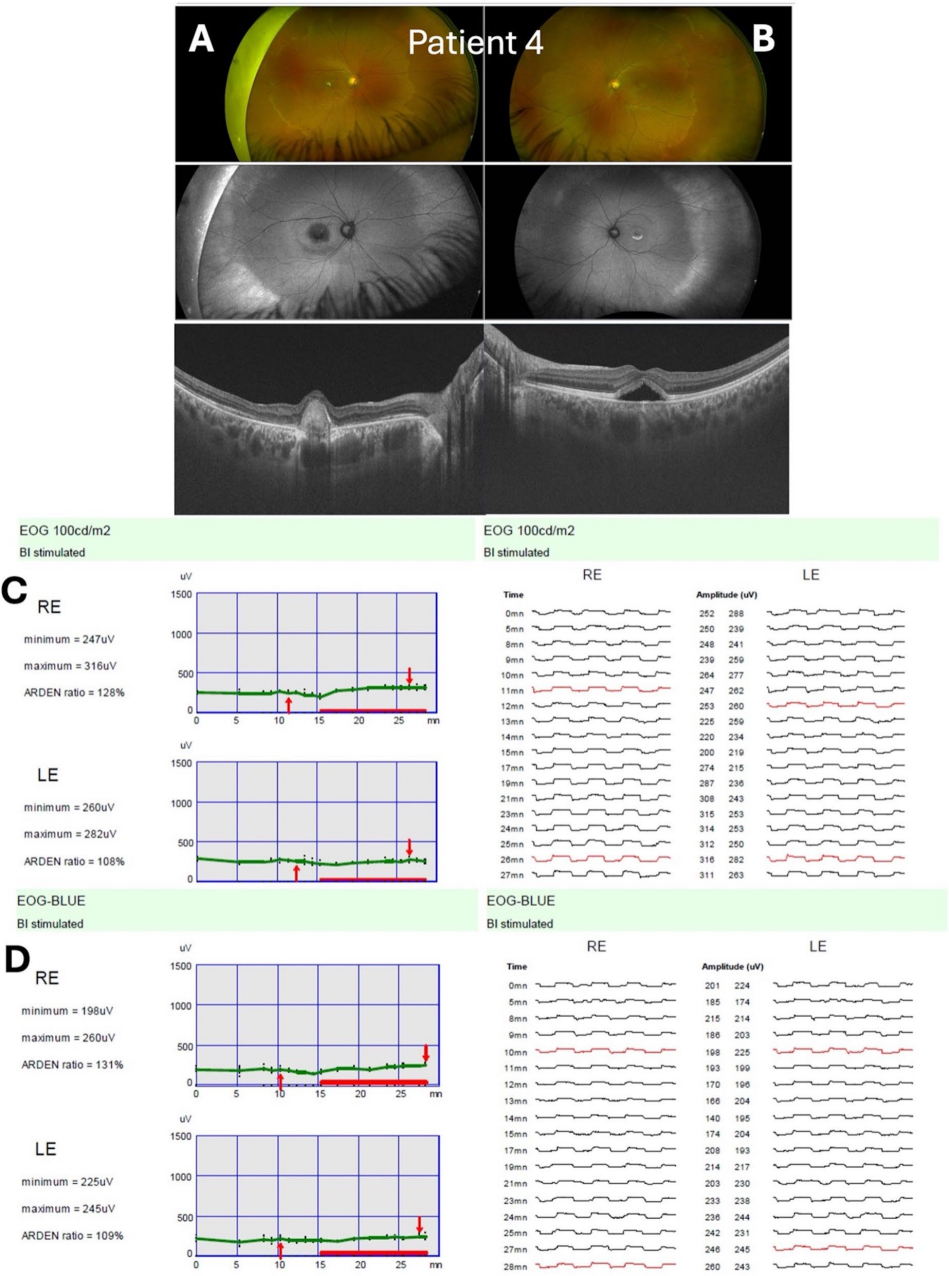
Patient 4 with Best disease. Right eye (vitelliform stage): Panel (**A**) shows the color fundus, fundus autofluorescence, and OCT images with a vitelliform lesion at the macula. Left eye (pseudohypopyon stage): Panel (**B**) illustrates the corresponding multimodal imaging with pseudohypopyon changes. Panels (**C**) and (**D**) demonstrate the ISCEV standard white light EOG (100 cd/m^2^) and SW-EOG (30 cd/m^2^ LED, 448 nm peak emission), respectively. Both eyes reveal reduced responses, with ‘Arden’ LP: DT_ratios_ reported for the RE and LE. A *BEST1* Exon 4 *c.287A* > *T (p.Gln96Leu*, heterozygous) variant was detected, classified as likely pathogenic

All four BVMD patients showed markedly reduced LP:DT_ratios_ under both white and SW stimulation (Table 1). The median LP:DT_ratio_ with white light was 1.30 (range 1.20–1.55), and with SW it was 1.28 (range 1.18–1.50). By comparison, age-matched normal individuals had ratios ~ 2.49 (white) and 2.47 (448 nm) [8]. Three patients had ratios well below 1.5 in both conditions (e.g. 1.20 and 1.18), while one patient was borderline (1.55 white vs 1.50 SW). In every case, both eyes were abnormal: e.g. Patient 1 had LP:DT_ratio_ of ~ 1.30 (RE) and 1.28 (LE) with white, and 1.25/1.23 with the SW-EOG (each about 50% below normal). Importantly, SW stimulation did not normalize or artificially elevated any LP:DT_ratio_; all SW blue ratios remained as low or slightly lower than the white-light values. The time to light peak was similar between stimuli (generally ~ 9–10 min for white vs ~ 8–9 min for SW) and showed no consistent trend. Figures 2–5 illustrate the clinical features of the patients alongside their ISCEV standard 100 cd/m^2^ white and SW-EOG responses. The testing times were equivalent for the SW-EOG and ISCEV standard EOG. Best Distance Corrected Visual Acuities (logMAR) for right eye (RE) and left eye (LE) and near vision for each patient were: Patient 1 RE 0.30, N6 and LE 0.60, N8; Patient 2 RE 0.20, N6 and LE 0.50, N10; Patient 3 RE 0.00, N6 and LE 0.00, N6, and Patient 4 RE 1.00, < N36 and LE 0.20, N12. Figure 1 shows a normal ISCEV 100 cd/m^2^ white and 30 cd/m^2^ SW-EOG recorded in a healthy individual from the site to illustrate the close relationship between the test protocols.

**Table 1:** EOG findings in four BVMD patients recorded with a white 100 cd/m^2^ or 30 cd/m^2^ 448 nm SW stimulus. LP:DT = light-peak:dark-trough ratio. All values were below normal (< 1.85) and in the diagnostic range for Best’s. Time to peak is in minutes. RE: Right eye; LE: Left eye

Patient	White LP:DT_ratio_	SW LP:DT_ratio_	Time to peak (White/SW)
1	1.30 (OD), 1.28 (LE)	1.25 (RE), 1.23 (LE)	Absence of light peak in both
2	1.45 (RE), 1.40 (LE)	1.42 (RE), 1.38 (LE)	Absence of light peak in both
3	1.55 (RE), 1.50 (LE)	1.50 (RE), 1.45 (LE)	Absence of light peak in both
4	1.20 (RE), 1.18 (LE)	1.18 (RE), 1.15 (LE)	Absence of light peak in both

Regarding comfort, all patients reported less discomfort with the SW light. On average, the discomfort score dropped from 3.5 (white) to 1.5 (SW) out of 5. Several patients noted that the white Ganzfeld (100 cd/m^2^) was “bright and harsh after dark adaptation,” whereas the SW (30 cd/m^2^) felt “dimmer and easier” with all participants rating the SW as more comfortable than the white stimulus (Fig. 2).

## Discussion

This series confirms that the SW-EOG reproduces the classic abnormal EOG in BVMD, without compromising diagnostic yield. All patients showed profoundly subnormal ‘Arden’ LP:DT_ratios_ (< 1.6) with either stimulus, consistent with Best’s disease. The LP:DT_ratios_ were essentially unchanged by the stimulus (white/SW) (e.g. Patient 1: 1.30/1.25; Patient 3: 1.55/1.50). These values align with prior reports (mean LP:DT_ratio_ of ~ 1.40 in pediatric Best’s) and differ markedly from normal individuals (~ 2.45) [5]. Thus, using the SW monochromatic light did not mask or alter the “light-rise defect”—the reduced RPE standing potential characteristic of BVMD [2, 3].

These findings support and extend the conclusions of Constable and Kapoor [8] to a disease context. In normal subjects, they showed virtually identical LP:DT_ratios_ for 100 cd/m^2^ white vs 30 cd/m^2^ SW light. In BVMD, we observed near-equivalence in the depressed ratio. The slight tendency for SW-EOG LP:DT_ratios_ to be marginally lower was not systematic enough to affect interpretation. In practice, all patients remained well below the diagnostic threshold under both conditions. Thus, diagnostic accuracy appears uncompromised by using 448 nm stimuli. Any small shifts in timing (white light peaks were, on average, 1–2 min later) are unlikely to change the clinical decision, since diagnosis depends primarily on the magnitude of LP:DT_ratio_ reduction [7, 22].

Importantly, patients uniformly preferred the SW stimulus. The lower photopic luminance and narrower spectrum produce less glare and photophobia, which is valuable given the prolonged fixation required by the test. In our series, no subject reported overt discomfort under the SW light, whereas the white Ganzfeld provoked squinting or complaints in all four. Improved patient comfort could enhance test reliability (less blinking, fatigue) and make EOG more feasible in sensitive populations (children, photophobic patients). Given that the clinical EOG already requires 15 min dark adaptation, reducing light discomfort is a practical benefit (Fig. 3).

From a methodological standpoint, adopting a monochromatic LED could streamline EOG setups. Many modern Ganzfeld devices support narrowband stimuli. However, wide adoption would require establishing new normative ranges for the 448 nm protocol in healthy subjects. Constable and Kapoor [8] provided a median value for the SW-EOG’s LP:DT_ratio_ of 2.47, suggesting that the diagnostic cutoff might remain similar (i.e. < 1.8). Our cases indicate that all Best patients fell well below any plausible threshold with the SW stimulus. Nonetheless, multicenter studies would be needed to confirm equivalence of diagnostic criteria (Fig. 4).

The EOG light-rise originates in RPE ion transport and is modulated by photoreceptor activity [10]. In Best disease, bestrophin-1 dysfunction disrupts RPE chloride conductance and calcium signaling, blunting the normal RPE’s basolateral membrane depolarization and the “light-rise” [23–25]. Our results suggest that a 448 nm stimulus is fully adequate to trigger this abnormal RPE response. Rods, which have peak sensitivity near 507 nm, still respond appreciably at 448 nm, and cones contribute as well. Since bestrophinopathy spares the photoreceptors themselves (full-field ERGs were normal in all our patients), any wavelength that elicits a robust photic drive will reveal the attenuated RPE potential (Fig. 5).

Clinical Implications: These findings indicate that using a 448 nm, 30 cd/m^2^ stimulus in place of the standard white Ganzfeld is feasible for BVMD workup. It reproduces the expected abnormal EOG signature of BVMD while enhancing patient tolerance. Clinicians might consider incorporating a SW-EOG protocol, especially for patients who struggle with bright lights. In future practice, if SW stimuli were standardized (with age-adjusted normal values), this could become an alternative ISCEV-recommended EOG protocol. In the interim, awareness of this option allows electrophysiologists to tailor testing. In addition, other suggestions for reducing the test time of the EOG include performing EOG recordings as part of the dark and light adaptation phases whilst recording the ISCEV full field ERGs as a screening tool [26]. EOGs recorded with a shortened dark and light adaption phase have also been suggested as alternatives to the standard ISCEV standard EOG recording protocol to reduce patient discomfort [27].

One caveat is the need for consistency: if switching stimuli, laboratories must recalibrate their “normal” LP:DT_ratio_. Nonetheless, as shown here and reported previously [8] the actual numerical change appears minimal. In our small series, there was no case where a patient’s diagnosis would have differed between stimuli. Larger studies should confirm that even borderline cases remain correctly classified. Finally, while the SW-EOG improves comfort, clinicians should note that it has lower overall illuminance; thus, it must be delivered in a true Ganzfeld to fully light-adapt the retina. One limitation is the small initial sample reported and future studies in larger cohorts of patients with BVMD, autosomal recessive, adult onset vitelliform macular dystrophy and Autosomal Dominant Vitreoretinochoroidopathy are planned to verify the clinical utility of the SW-EOG. The pathogenic mutations in this series of patients were all *BEST1* it will be of interest to determine if similar results are obtained with disease causing variants in genes such as *PRPH2* [28] and *IMPG1, IMPG2* [29, 30].

## Conclusions

In summary, this case series demonstrates that the SW-EOG elicits the same profound EOG light-rise deficit in BVMD as standard white light. All Best patients retained severely reduced LP:DT_ratios_ under both conditions, confirming the diagnosis in each case. Crucially, the SW 30 cd/m^2^ stimulus was substantially more comfortable, supporting its potential clinical use. These results, together with recent normative studies, suggest that a 448 nm LED could effectively substitute for the standard white Ganzfeld in EOG testing, without loss of diagnostic information. Electrophysiologists should consider the implications for patient care: improved comfort and equivalent diagnostic performance. Further research with larger cohorts and in other bestrophinopathies will help establish guidelines for integrating spectral stimuli into routine EOG practice.

## Data Availability

Not applicable.
